# A Review of Three Simple Plant Models and Corresponding Statistical Tools for Basic Research in Homeopathy

**DOI:** 10.1100/tsw.2010.224

**Published:** 2010-12-14

**Authors:** Lucietta Betti, Grazia Trebbi, Michela Zurla, Daniele Nani, Maurizio Peruzzi, Maurizio Brizzi

**Affiliations:** ^1^ Department of Agroenvironmental Sciences and Technologies, University of Bologna, Italy; ^2^ Italian Society of Anthroposophical Medicine, Milan, Italy; ^3^ Association for Sensitive Crystallization, Sondrio, Italy; ^4^ Department of Statistical Sciences, University of Bologna, Italy

**Keywords:** wheat, germination, growth, tobacco plants, tobacco mosaic virus, Poisson test, Mann-Whitney test, Wilcoxon test

## Abstract

In this paper, we review three simple plant models (wheat seed germination, wheat seedling growth, and infected tobacco plants) that we set up during a series of experiments carried out from 1991 to 2009 in order to study the effects of homeopathic treatments. We will also describe the set of statistical tools applied in the different models. The homeopathic treatment used in our experiments was arsenic trioxide (As_2_O_3_) diluted in a decimal scale and dynamized. Since the most significant results were achieved with the 45th decimal potency, both for As_2_O_3_ (As 45x) and water (W 45x), we here report a brief summary of these results. The statistical analysis was performed by using parametric and nonparametric tests, and Poisson distribution had an essential role when dealing with germination experiments. Finally, we will describe some results related to the changes in variability, which seems to be one of the targets of homeopathic treatment effect.

### INTRODUCTION

The effectiveness of homeopathy is still an open topic in the scientific community, and the debate has recently been taken up by a meta-analysis published in *The Lancet*[1]. In fact, after this publication, fundamental methodological problems about the above-cited meta-analysis have been detected[2,3,4,5]. Homeopathic preparations, also called “potencies”, are produced by diluting and rhythmically succussing a mother tincture (“potentization process”). At ultramolecular dilutions (beyond the Avogadro limit), the probability of the presence of molecules of the original substance is near to zero[6]. Therefore, most criticism concerns the specific effects of these very high dilutions, which are judged to be “implausible” according to conventional science, although there is emerging evidence for their *in vitro*[7,8,9,10, 11,12,13,14] and *in vivo*[15,16,17,18,19,20,21,22,23,24,25,26,27] activity. For homeopathy to become

Since one of the most repeated criticisms about the efficacy of homeopathic preparations is the presence of a placebo effect, a suitable way to overcome this objection is to define experimental models where "patients" are not human beings, but plants or microorganisms instead[47,48,49]. Furthermore, relatively simple model systems have the advantage of a more direct treatment/effect relationship, and give the opportunity of collecting large data samples for structured statistical analyses, particularly important when dealing with ultramolecular dilutions. Moreover, in such a context, characterized by research patterns where new possible ways of investigation may be opened at any time and little previous literature is available, the choice of suitable statistical tools has to be reconsidered carefully for every series of experiments. The presence of a professional statistician within research groups in homeopathy and other complementary medicines is strongly recommended, and in the past 10 years, several European research groups have published some excellent papers on studies in homeopathy using high-quality, elegant, and advanced statistical methods[4,5,22,23,24,25,26]. We think that research work based on plant models, if it gives the appropriate weight to statistical analysis and interpretation of results, could yield important contributions to determine the effectiveness of homeopathic treatments. Thus, the aim of this review was to report the most important results obtained during our series of experiments (1991–2009) on different plant models that we set up. Specifically, we considered wheat germination and growth, as well as tobacco/tobacco mosaic virus (TMV) interaction, giving a detailed description of the statistical methods applied. Due to the specific biological features of the above-mentioned plant models, characterized by specific working variables, the statistical approach was necessarily differentiated. In particular, we review all the results concerning the 45$^{th}$ decimal potency (45x), both for arsenic trioxide (As 45x) and water (W 45x), since it yielded the most significant and reproducible results for every kind of model.

### PRODUCTION OF HOMEOPATHIC PREPARATIONS

Potentized arsenic (As 45x) was obtained through serial dilution (1:10) with pure water (p.A., Merck, Darmstadt, Germany) and succussion, starting from a 0.20% solution (0.01 M) of arsenic trioxide ($As_2O_3$, Aldrich, St. Louis, MO, USA). The dynamization was performed using a specially designed succussion machine that vertically shakes 1000-ml volumes (in polyethylene bottles filled to 90% of capacity) at a rate of 70 times per minute with an oscillation amplitude of 24 cm; each potency was succussed for 1 min. Potentized water (W 45x) was prepared using exactly the same method of serial dilution and succussion, with the only difference being that the starting solution was the solvent (pure water, Merck). Pure water (p.A., Merck) was also utilized as a control (C). The different treatments were then poured into polyethylene bottles and letter coded according to a blind protocol by a person not involved in the experiments. In order to reduce microbial growth, bottles were stored at a cool temperature (4°C) until use[13].

### *IN VITRO* WHEAT GERMINATION

#### Biological Protocol

The first and most simple model we used is based on *in vitro* wheat (*Triticum aestivum* L.) germination[7,8,13]. In this model, a sample of seeds was placed on sterilized sand in randomly distributed Petri dishes (Fig. 1A, B). Since homeopathic treatments in human medicine are to be used in unhealthy individuals, we tried to reproduce a similar "disease pattern" by adopting an isopathic approach.

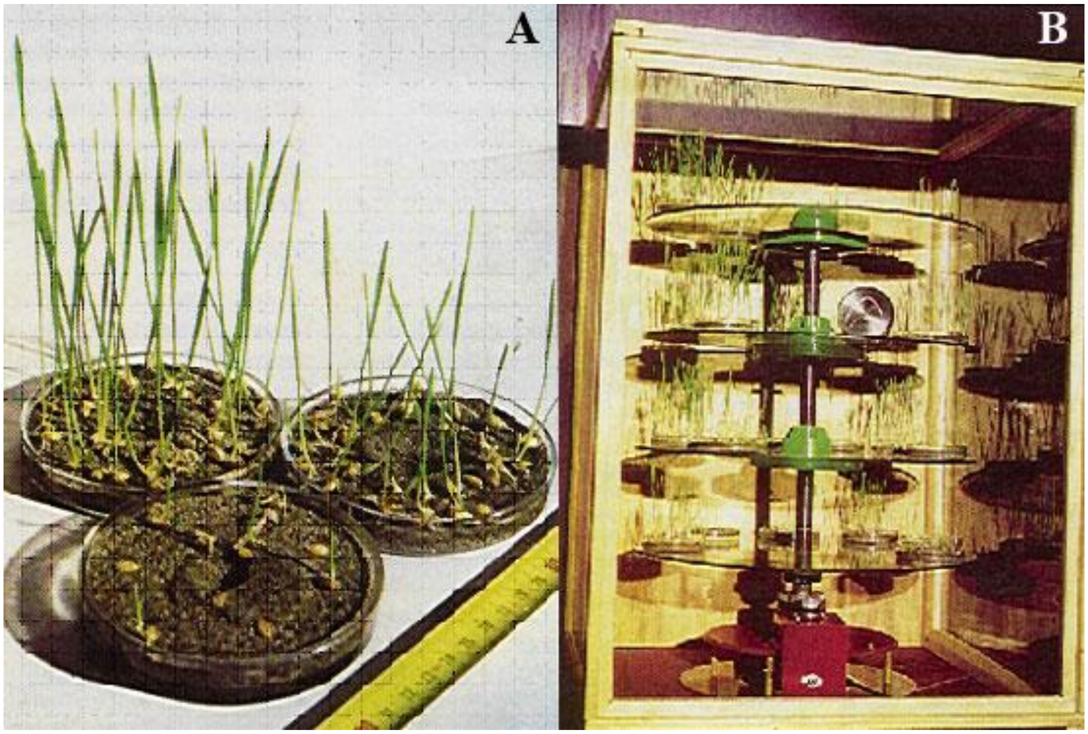

**FIGURE 1**. Wheat germination model. (A) Seeds placed on sterilized sand in Petri dishes; (B) Petri dishes randomly distributed in a germination box.

According to "isopathy", the same substance causing the disease can be used in low doses or high dilutions to treat the disease itself[20]. This concept is analogous to the hormetic effect[50,51]. Therefore, in some of our experiments[8,13], we previously stressed wheat seeds with ponderal sublethal doses of $As_2O_3$[52], thus reducing germination rate (see methodology in Brizzi et al.[13]).

Stressed seeds were then treated with a fixed quantity of treatment: $As_2O_3$ diluted and dynamized at the *k*-th decimal potency, for several values of *k* ranging from 23 to 45. The other treatment groups were potentized water (W 23 to 45x) and pure water (C). The working variable is the number of nongerminated seeds per dish out of a fixed number of 33 seeds, after 4 days of observation. Here we will report only the results obtained with 45x potency (As 45x and W 45x).

### Statistical Analysis: Poisson Distribution

At the beginning of our series of experiments, we realized that Poisson distribution fits the number *X* of nongerminated seeds per Petri dish very satisfactorily. Just to give an example, we report here (Fig. 2) the graphical comparison of empirical and Poisson cumulative distribution function for the control group[7]; we can clearly note that theoretical and empirical values are very close. Nevertheless, we repeatedly checked the goodness-of-fit by means of the Kolmogorov-Smirnov test[53], applied to different experimental samples (both control and treatment groups), and we never detected a significant difference. We also confirmed the adequacy of Poisson distribution for wheat germination data with a graphical method proposed by Hoaglin[54], called the *Poissonness plot*. If we denote the values of the working variable *X* with *x* ($x$ = 0, 1, 2, …) and the corresponding frequencies with *n(x)*, in a two-dimensional diagram, we can plot *x* values against

$$y(x) = \ln n(x) + \ln (x!) \qquad (1)$$

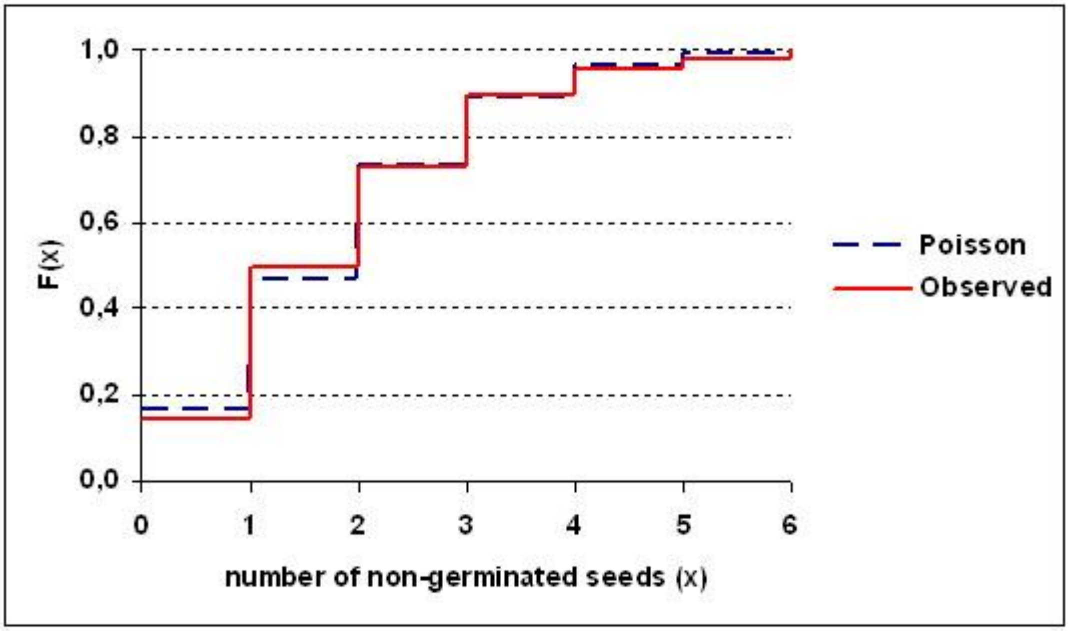

**FIGURE 2.** Empirical and Poisson distribution function (control group); goodness-of-fit of Poisson model to wheat germination data.

If the plotted points approximately follow a straight line, the Poisson distribution fits well. We checked the linearity of the plotted points by the Bravais-Pearson correlation coefficient *r(x,y)*. Since the *r* values calculated are all greater than 0.83, we went on supposing that *X* follows a Poisson distribution under all the experimental situations we dealt with.

Once the goodness-of-fit of the Poisson model to wheat germination data had been demonstrated, we were able to apply the specific parametric tests for this distribution. When doing pairwise comparisons, where the null hypothesis is $H_0$: $\lambda_A = \lambda_B$, i.e., the equality of two Poisson parameters, if the samples have the same size, we can apply the following test statistic, reported by Sachs[55], denoted here with $\hat{z}$ :

$$\hat{z} = \frac{T_A - T_B - 1}{\sqrt{T_A + T_B}} \tag{2}$$

where $T_A$ and $T_B$ are the total number of nongerminated seeds in the two samples compared. Under the null hypothesis $H_0$, the test statistic (Eq. 2) follows a standard normal distribution.

Since in most of our experiments control groups (C) were larger than treatment groups (T), when comparing C vs. T, we applied an exact Poisson test in the following way: if $\hat{\lambda}_C$ is the maximum likelihood estimate of the Poisson parameter in the control group, and $n_T$ is the sample size in the treatment group, we calculate the Poisson probabilities with parameter $\hat{\lambda} = \hat{\lambda}_C \cdot n_T$, which corresponds to treatment distribution under the null hypothesis $\lambda_C = \lambda_T$. Therefore, given a significance level α, if the observed number of nongerminated seeds in the treatment group, say $N_T$, lies in the tails of the Poisson distribution with parameter $\hat{\lambda}$, we rejected the null hypothesis.

When comparing more than two Poisson distributions in a global comparison, in a sort of "Poissonian ANOVA", we applied the test proposed by Sachs[55]. The null hypothesis is now $H_0$: $\lambda_1 = \lambda_2 = \ldots = \lambda_k$ ; we used $x_i$ to denote the total number of nongerminated seeds in the *i*-th sample, with $t_i$ the total number of experiments of the same sample,

$$\hat{\lambda} = \frac{\sum_{i=1}^{k} \hat{\lambda}_i \cdot t_i}{\sum_{i=1}^{k} t_i} \quad (3)$$

being the overall Poisson parameter estimate. Now, if we compute the values:

$$z_i = \begin{cases} 2(\sqrt{x_i + 1} - \sqrt{t_i \cdot \hat{\lambda}}) & if \quad \hat{\lambda}_i < \hat{\lambda} \\ 2(\sqrt{x_i} - \sqrt{t_i \cdot \hat{\lambda}}) & if \quad \hat{\lambda}_i \geq \hat{\lambda} \end{cases} \quad (4)$$

the overall Poisson test statistic is the following:

$$w = \sum_{i=1}^{k} z_i^{\,2} \cong (H_0) \cong \chi^2_{k-1} \quad (5)$$

The test is one-tailed on the right, so we reject $H_0$ only for large values of *w*.

When dealing with multiple treatments, we began our comparison with the global test based on the statistic *w*; after checking that overall comparison was significant, we went on with pairwise tests.

### Main Results

First, we compared control samples with a global Poisson test and the results were never significant; a comparison between five independent experiments at the same temperature yielded a *w*-statistic value (see Eq.5) of 4.51, very far from significance[13]. This finding gives us important methodological information, assuring us that our model is stable and that observed differences are to be imputed to treatment effect. This was confirmed by the fact that the global Poisson test becomes significant when adding treatment groups. Although we considered several decimal potencies in these experiments, we report here only the results referring to As 45x and W45x with respect to the control. It should be remembered that our working variable is the number of nongerminated seeds in a “standard trial” of 33 seeds in a Petri dish.

Looking at Table 1, we can immediately note that control mean values seem to be rather regular throughout our period of experimentation, both for nonstressed and stressed seeds. This induces us to believe that our model is quite stable and that observed differences can be properly attributed to treatment effect. Treatment with dynamized arsenic and water does induce a significant reduction in the number of nongerminated seeds, showing a stimulating effect (except for nonstressed seeds treated with W 45x in the 1st experiment, 2000[8]). Such an effect is considerably more significant (*p* value constantly less than 0.01) and always reproducible when using As 45x. Confirming our forecasts, the treatment effect was more evident when working with stressed seeds; this is the reason for using only stressed seeds in the last experiment[13]. Finally, to simplify the visual impression and interpretation of our results, in Fig. 3 we present the average values of germinated seeds normalized against the corresponding control values, set equal to 100; here, the As 45x treatment class has the most noticeable effects, with a germination increase up to 12% in stressed seeds.

**TABLE 1**
**Wheat Germination Model: Mean Number of Nongerminated Seeds and Poisson Test Significance**

| Reference | Stress | n | Control | As 45 x | | W 45 x | |
|---|---|---|---|---|---|---|---|
| | | | Mean | Mean | *p* Value | Mean | *p* Value |
| (a) Betti et al. (1994) | No | 24 | 1.79 | 1.04 | 0.0021 (**) | — | — |
| (b) Brizzi et al. (2000) 1st exper. | No | 24 | 1.54 | 0.94 | 0.0056 (**) | 1.44 | n.s. |
| (c) Brizzi et al. (2000) 2nd exper. | No | 24 | 2.00 | 1.13 | 0.0042 (**) | 1.13 | 0.0042 (**) |
| (d) Brizzi et al. (2000) 1st exper. | Yes | 24 | 5.03 | 3.25 | <0.0010 (***) | 3.78 | <0.0010 (***) |
| (e) Brizzi et al. (2000) 2nd exper. | Yes | 24 | 6.58 | 3.38 | <0.0010 (***) | — | — |
| (f) Brizzi et al. (2009) | Yes | 48 | 5.71 | 4.50 | 0.0044 (**) | 4.90 | 0.0419 (*) |

Legend: n = number of standard trials; the *p* values are related to comparisons with control; n.s. = not significant; *$p \leq 0.05$; **$p \leq 0.01$; ***$p \leq 0.001$.

Data for this table are taken from earlier publications: (a) [7]; (b–e) [8]; (f) [13]. In the paper [8], we described two separate experiments, here denoted with 1st and 2nd; in the paper [13], we reported data referring to different temperatures, here we report only those obtained at 20°C.

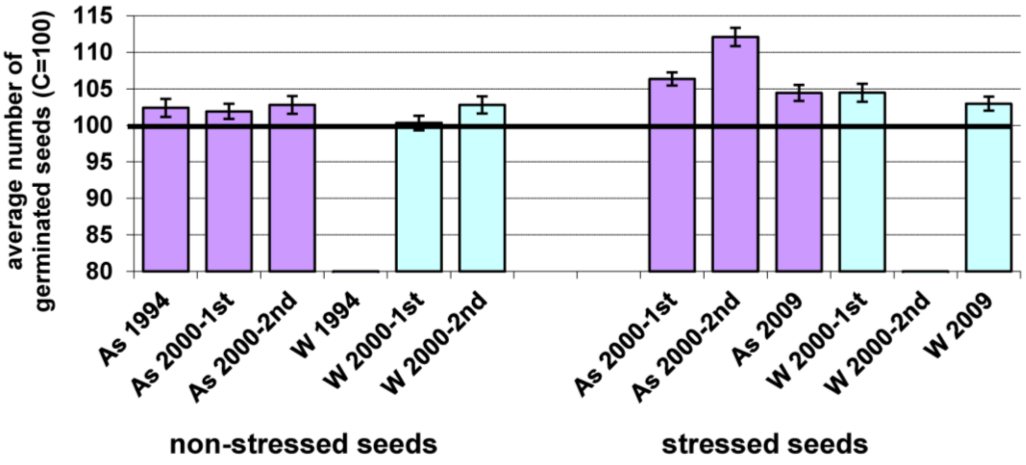

**FIGURE 3**. Average number of germinated seeds in each experiment (control = 100). Bars indicate standard errors of average values.

### *IN VITRO* WHEAT GROWTH

#### Biological Protocol

Using the same plant species, we set up a second isopathic model (*in vitro* wheat growth), where a different biological parameter was evaluated[15,19]. In fact, in this model, the working variable considered is the stem length Y(d) of wheat seedlings measured after d days from seeding (d = 4, 5, 6, 7). We considered Y(7) as the main variable, i.e., the stem length on day 7, assigning a value of "0" to nongerminated seeds. In this kind of experiment, each seed was placed in a transparent cellophane envelope, inserted in a larger cardboard envelope, so that stem and roots could develop in natural light and darkness, respectively (Fig. 4). In this model, stressed seeds were treated with a fixed quantity of homeopathic preparations and here we will report only the results obtained with 45x potency (As 45x and W 45x) with respect to the control (C).

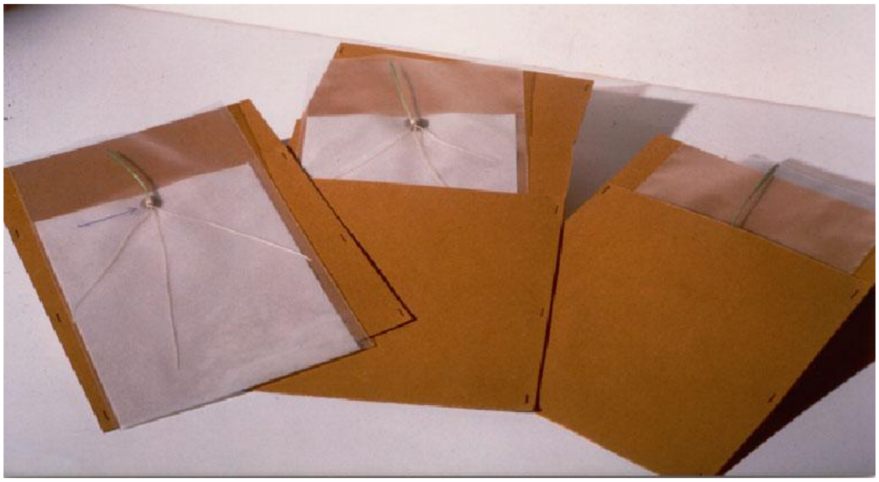

**FIGURE 4**. Wheat growth model; seed attached to filter paper by a piece of clay, then placed in a transparent cellophane envelope and inserted in a larger cardboard envelope.

### Statistical Analysis: Student's t and Mann-Whitney Tests

In the first wheat growth experiment[15], we decided to apply the classic Student's *t*-test because our sample was very large (150 data for each of the two treatment groups, C and As 45x) and the distribution seemed to be unimodal and not so far from symmetry (checked by means of Pearson's index). On the other hand, in the second experiment[19], the samples were considerably smaller because we checked five different potencies simultaneously (5, 15, 25, 35, and 45x) and it proved to be more skewed, so we preferred to apply a nonparametric test. Indeed, whenever we are dealing with skewed data, which are not to be presumed as normally distributed, we need to choose specific tools to make statistical inferences. In particular, we applied the Mann-Whitney rank sum test (U-test) here, where the null hypothesis states that two populations (treated and control) have the same level of magnitude (see, e.g., Conover[56]). Briefly, this test is based on a global ranking of sample data; if the ranks of data from one sample are considerably smaller or greater than the other sample's ranks, the null hypothesis is rejected. The test statistics U is based on the sum of ranks of each sample; when the sample is small, we do need specific tables, but if the sample is not too small (at least 20 observations in each sample), we can use a normal approximation.

### Main Results

Looking at Table 2, it is easy to note that the As 45x treatment always induced a significant stimulating effect on wheat seedling growth. We can also observe that the percentage stimulating effect is almost the same (+24.0 and +24.7%, respectively), even if the control mean is considerably different in the two experiments due to seasonal effect (the first was carried out in winter, the second in summer). Regarding dynamized water (W 45x), we did not detect any significant effect in this model, although there was an increase of almost 20% in mean stem length, as reported in Table 2. When comparing the average daily increase observed for As 45x in the first stem length experiment (1997[15]) with that observed in the second one (2005[19]), the results prove to be very close and in both experiments an almost perfectly linear growth trend can be observed (Fig. 5).

**TABLE 2**
**Wheat Growth Model: Mean Stem Length (cm) and Mann-Whitney Test Significance**

| Reference | Stress | n | Control | As 45 x | | W 45 x | |
|---|---|---|---|---|---|---|---|
| | | | Mean | Mean | *p* Value | Mean | *p* Value |
| (g) Betti et al. (1997) | Yes | 150 | 3.17 | 3.94 (+24.0%) | 0.001 (***) | — | — |
| (h) Brizzi et al. (2005) | Yes | 30 | 6.02 | 7.51 (+24.7%) | 0.042 (*) | 7.22 (+19.9%) | n.s. |

Legend: n = number of seedlings; the *p* values are related to comparisons with control; n.s. = not significant; *$p \leq 0.05$; ***$p \leq 0.001$.

Data for this table are taken from earlier publications [15,19].

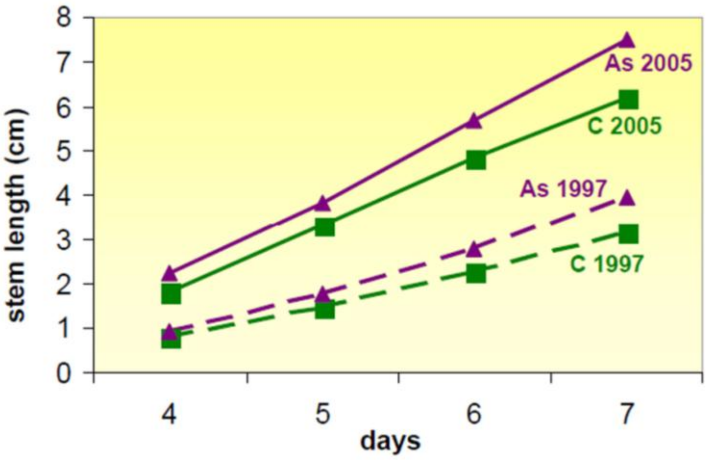

**FIGURE 5.** Time trend of the effect on the stem length of As 45x (As) vs. control (C); comparison between the original trial (1997, dotted line) and second trial (2005, continuous line). C is represented by squares and As by triangles.

## TOBACCO/TOBACCO MOSAIC VIRUS INTERACTION

### Biological Protocol

The effects of homeopathic treatments were also checked on a well-known phytopathological model, tobacco plants (*Nicotiana tabacum* L. cultivar Samsun), bearing the TMV-resistance gene *N*, subjected to TMV inoculation as biotic stress[16,48]. These resistant plants react to the virus with the so-called hypersensitive response (HR), a typical plant defense mechanism that occurs in resistant plants in response to different stresses. HR is defined as a rapid, localized programmed cell death, which results in the formation of foliar necrotic lesions around the infection sites, and is associated with restricted pathogen multiplication and spread[57,58,59]. The higher the host resistance, the quicker the defense response, expressed by fewer and smaller necrotic lesions. As there is no systemically compiled *Materia Medica* for plants, it is not possible to approach diseased plants by the classical homeopathic remedy selection procedure and, thus, different methods can be applied[48]. In particular, in the tobacco/TMV model, the "remedy" was selected according to the *law of similars* ("like cures like")[60], i.e., when a substance in a high dose is able to induce defined symptoms in a healthy living system, it is also able to cure these symptoms when applied in a low or very low dose. Therefore, homeopathic treatments were chosen on the basis of the hypersensitive-like reaction (necrotic spots) induced by $As_2O_3$ in phytotoxic concentration (1 mM) on tobacco leaves[48,59]. These lesions resemble those provoked by TMV during a hypersensitive response, both phenotypically (Fig. 6) and histochemically (Fig. 7, see methods in Ruzin[61] and Johansen[62]). Experiments were performed in a greenhouse under controlled conditions and a purified TMV-type strain suspension was used for virus inoculation. A large number of leaf disks from TMV-inoculated plants were placed for 3 days in randomly distributed Petri dishes containing the same quantity of pure water (C), W 5x or 45x, As 5x or As 45x (Fig. 8). The working variable here is the number and area of hypersensitive lesions (necrotic spots) observed in a leaf disk 3 days after virus inoculation[16,48,63]. Digital images of inoculated leaf disks were acquired by means of a flatbed scanner (Epson Perfection 2480 Photo) and utilized for lesion area assessment, using the Assess software[64]. The same disks were then placed on a transilluminator for lesion number counting. The research was structured in eight separate experiments; in the present paper, we report the results of the three experiments involving W 45x and As 45x.

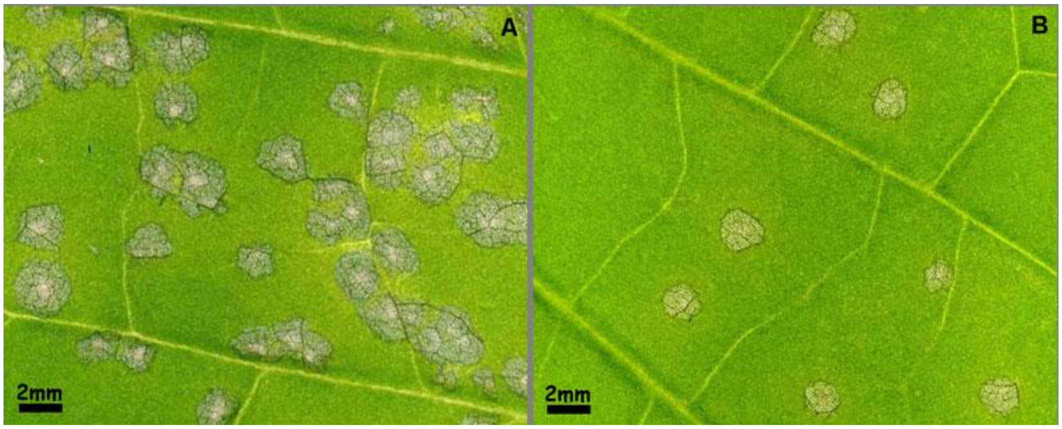

**FIGURE 6.** Principle of similarity in tobacco leaves inoculated with TMV; hypersensitive lesions induced by TMV (A) and necrotic spots induced by $As_2O_3$ in phytotoxic concentrations (B).

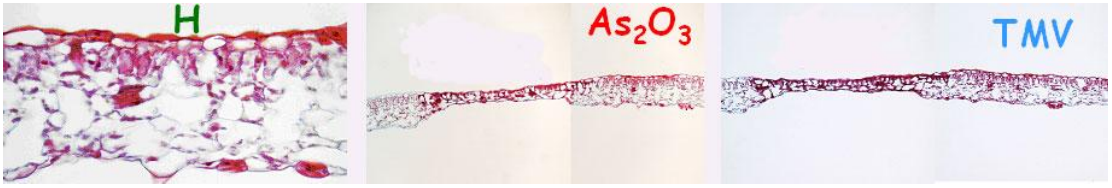

**FIGURE 7.** Cross-sections of tobacco leaves; H = healthy leaf, $As_2O_3$ = leaf treated with 1 mM $As_2O_3$, TMV = leaf inoculated with TMV.

### Statistical Analysis: Nonparametric Tests Based on Ranks

Since the data were evidently skewed, we used the nonparametric Wilcoxon rank sum test in this case. In this test, we needed to calculate the differences of paired values (unlike in the Mann-Whitney test, data have necessarily to be numerical here), assigning a rank to each difference; the null hypothesis is rejected when there is a prevalence of differences of the same direction with respect to the other direction. Observations of the two groups were paired using a criterion based on cograduation. This test needs specific tables that are easily available (also on the web). We applied the Wilcoxon test both to the number and area of hypersensitive lesions.

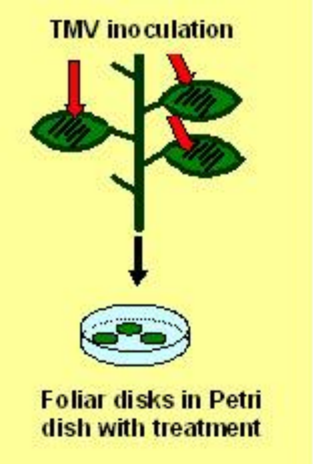

**FIGURE 8.** Tobacco/TMV model; schematic representation of the experimental protocol.

Since there might be some criticisms about the pairing criterion based on cograduation, we also applied the Mann-Whitney test (for independent samples) to the same data.

### Main Results

As far as the mean number of hypersensitive lesions is concerned, the results are summarized in Table 3. It can be seen that both treatments (As 45x and W 45x) induced a highly significant mean reduction with respect to the control, although there is an exception for dynamized water in the third experiment, where the number of necrotic lesions were larger than in the control group[16]. Fig. 9 reports the overall means of both lesion number and area (with respect to the control equal to 100); a highly significant decrease of both parameters can be observed. This effect can be related to an improvement of plant resistance due to homeopathic treatment. The reduction in the number and area of hypersensitive lesions in TMV-inoculated leaf disks can be observed in Fig. 10. The significant results obtained with W 45x (Table 3 and Fig. 10) suggest that solvent dynamization alone is able to induce effects similar to, but weaker than, homeopathic arsenic, as seen in the wheat germination model in Table 1. It is worth pointing out that by applying the Mann-Whitney test, we again obtained highly significant results.

**TABLE 3**
**Tobacco/TMV Interaction: Mean Number of Necrotic Lesions and Wilcoxon Test Significance**

| Reference | n | Control | As 45 x | | W 45 x | |
|---|---|---|---|---|---|---|
| | | Mean | Mean | *p* Value | Mean | *p* Value |
| (i) Betti et al. (2003) 1st exper. | 90 | 76.6 | 58.6 | <0.001 (***) | 68.7 | <0.001 (***) |
| (i) Betti et al. (2003) 2nd exper. | 90 | 118.2 | 81.4 | <0.001 (***) | 64.4 | <0.001 (***) |
| (i) Betti et al. (2003) 3rd exper. | 90 | 93.3 | 85.1 | <0.001 (***) | 108.8 | <0.001 (***) |
| (i) Overall experiment | 270 | 96.0 | 75.0 | <0.001 (***) | 80.6 | <0.001 (***) |

Legend: n = number of leaf disks; the *p* values are related to comparisons with control; ***$p \leq 0.001$.

Data for this table are taken from an earlier publication [16].

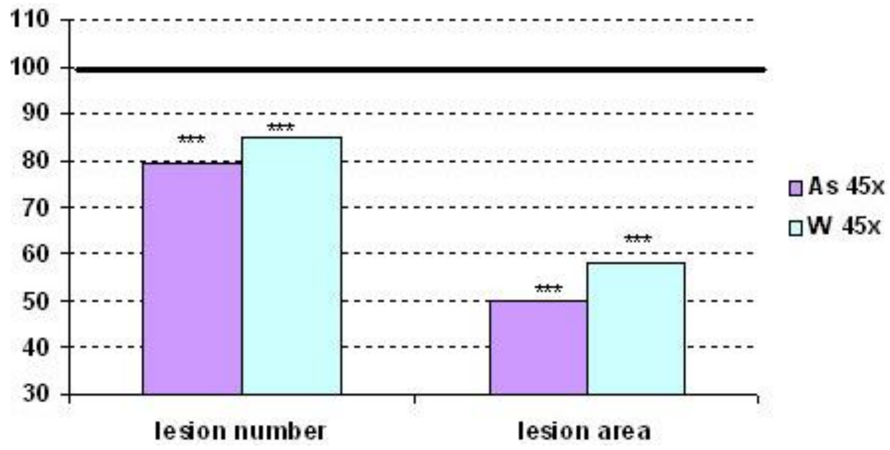

**FIGURE 9**. Tobacco/TMV model; hypersensitive lesion number and area induced by As 45x and W 45x (control = 100); *** ≤ 0.001.

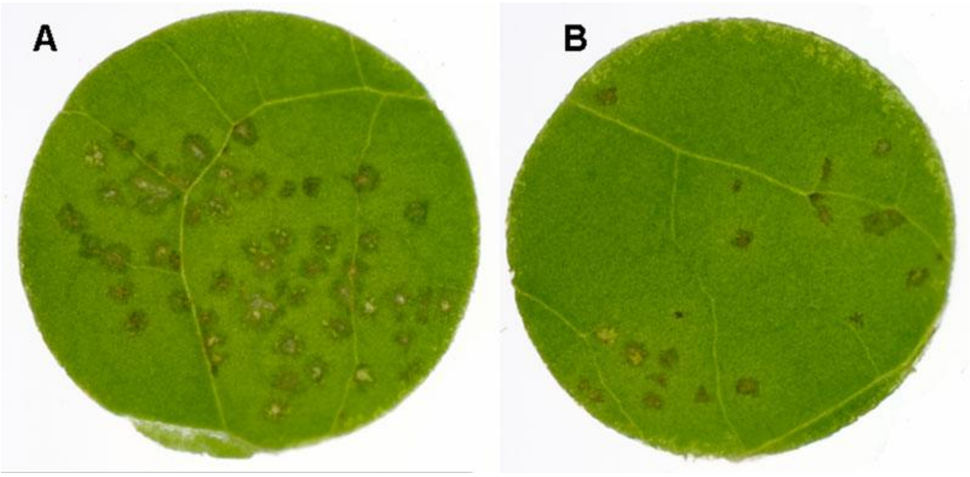

**FIGURE 10.** Hypersensitive lesions (necrotic spots) in tobacco leaf disks inoculated with TMV and treated with either pure water as a control (A) or As 45x (B); homeopathic treatment (B) induces fewer and smaller lesions with respect to control (A).

### VARIABILITY AND EFFECT SIZE

#### Theory and Methods

A further analysis was performed to check variability[16,65]. Indeed, we considered it not only as a useful tool for evaluating mean results, but as one of the markers for detecting the effects of homeopathic high dilutions. In fact, we observed a repeated and not negligible decrease in variability in treated groups for all the models we considered[65]. Variability was also tested and described by other research groups with not always concordant results[18,22,25].

Since the mechanism of action of homeopathic treatments is not at all clear, we tried to deepen our analysis by splitting variability into its two components, distinguishing variability "within" experiments from variability "between" experiments and making statistical comparisons for both components. If we indicate the number of experiments with $q$, the $k$-th observation of the $i$-th experiment with $y_{ik}$, the sample size and mean value of the *i*-th experiment with $n_i$ and $m_i$, respectively, *n* being the overall sample size and *m* the global mean value, we have:

$$SD \text{ (global)} = \sqrt{\frac{\sum_{i=1}^{q}\sum_{k=1}^{ni}(y_{ik}-m)^2}{n}} \qquad (6)$$

$$SD_W \text{ (within experiments)} = \sqrt{\frac{\sum_{i=1}^{q}\sum_{k=1}^{ni}(y_{ik}-m_i)^2}{n}} \qquad (7)$$

$$SD_B \text{ (between experiments)} = \sqrt{\frac{\sum_{i=1}^{q}(m_i-m)^2 n_i}{n}} \qquad (8)$$

We adopted standard deviation as a marker of variability because it has the same unit of measurement of data, but we have to consider that, due to the square root effect, the sum $SD_W + SD_B$ can no longer be exactly equal to *SD*. We calculated the classical F-test for comparing standard deviations; we also proposed a nonparametric approach, applying a simple sign test to the sign of the differences.

The effect size ("d") of treatments in the three models was computed as ratio between absolute difference (treatment – control) and standard deviation of control.

### Main Results

The overall standard deviation shows a trend towards a systematic decrease in treated groups, with just one exception with an almost immaterial increase (Table 4). The comparison of variances (F-test) is significant in four experiments (Table 4 [a], [e], [h], [i]) and near to significance ($p < 0.10$) in two others (Table 4 [b], [f]). Splitting the variability into its two mains components (Table 5), we can easily observe that variability between experiments (expressed by $SD_B$) in treated groups is smaller than in the control, in all the plant models we considered**.** If we apply the sign test based on binomial distribution, we reach a *p* value of 0.002 (highly significant), although the F-test shows a significant result ($p = 0.04$) only in the wheat germination model (Table 5 [e]). Moreover, variability within experiments (expressed by $SD_W$) is also generally smaller with only two exceptions (Table 5 [d], [g]): the F-test shows a significant difference in the wheat growth model (Table 5 [h]) with $p = 0.03$, and in the tobacco/TMV model (Table 5 [i]) with $p = 0.01$; in the wheat germination model, we have two nearly significant values with $p < 0.10$ (Table 5 [c], [f]). These findings suggest that homeopathic treatments sometimes have a significant effect on variability, and this kind of effect is worthy of a deeper study.

The standard deviation values allowed us to calculate also the effect size "d" of the treatments (As 45x and W 45x) in all three models (Table 6). It can be seen that "d" values are quite regular and relevant when comparing As 45x vs. control (particularly when considering germination of stressed wheat seeds). The same "d" value calculated for As 45x vs. W 45x shows heterogeneous results. Finally, the size effect of W 45x vs. control shows not negligible values, although reduced with respect to As 45x.

**TABLE 4**
**Global Standard Deviation Observed in Different Plant Models (Treatment vs. Control)**

| Plant Models | Stress | Standard Deviation | | | | |
|---|---|---|---|---|---|---|
| | | Control | As 45 x | Diff. % | F-Value | *p* Value |
| Wheat germination | | | | | | |
| (a) Betti et al. (1994) | No | 1.35 | 0.89 | -34.3 | 2.252 | 0.02 (*) |
| (b) Brizzi et al. (2000) 1st exper. | No | 1.33 | 1.03 | -22.8 | 1.670 | 0.07 (x) |
| (c) Brizzi et al. (2000) 2nd exper. | No | 1.58 | 1.27 | -19.8 | 1.548 | n.s. |
| (d) Brizzi et al. (2000) 1st exper. | Yes | 2.37 | 2.14 | -9.7 | 1.227 | n.s. |
| (e) Brizzi et al. (2000) 2nd exper. | Yes | 3.50 | 2.41 | -31.1 | 2.109 | 0.04 (*) |
| (f) Brizzi et al. (2009) | Yes | 2.47 | 1.86 | -24.8 | 1.763 | 0.07 (x) |
| Wheat growth | | | | | | |
| (g) Betti et al. (1997) | Yes | 1.98 | 2.03 | +2.4 | 0.951 | n.s. |
| (h) Brizzi et al. (2005) | Yes | 3.63 | 2.57 | -29.3 | 1.995 | 0.03 (*) |
| Tobacco/TMV | | | | | | |
| (i) Betti et al. (2003) | Yes | 59.99 | 51.18 | -14.7 | 1.374 | 0.003 (**) |

Legend: the *p* values are related to comparisons with control; n.s. = not significant; x = $p \leq 0.10$; *$p \leq 0.05$; **$p \leq 0.01$.

Data for this table are taken from an earlier publication [65].

**TABLE 5**
**Standard Deviation Components Observed in Different Plant Models**
**(W = Within Experiments, B = Between Experiments)**

| Plant Models | Stress | n | Std. Dev. (W) | | | Std. Dev. (B) | | |
|---|---|---|---|---|---|---|---|---|
| | | | Control | As 45x | Diff. (%) | Control | As 45x | Diff. (%) |
| Wheat germination | | | | | | | | |
| (a) Betti et al. (1994) | No | 16 | 1.04 | 0.60 (n.s.) | -42.3 | 0.86 | 0.65 (n.s.) | -24.3 |
| (b) Brizzi et al. (2000) 1st exper. | No | 12 | 1.12 | 0.86 (n.s.) | -23.4 | 0.71 | 0.56 (n.s.) | -21.4 |
| (c) Brizzi et al. (2000) 2nd exper. | No | 8 | 1.32 | 0.94 (x) | -28.6 | 0.87 | 0.85 (n.s.) | -2.4 |
| (d) Brizzi et al. (2000) 1st exper. | Yes | 12 | 1.32 | 1.46 (n.s.) | +10.8 | 1.97 | 1.56 (n.s.) | -20.6 |
| (e) Brizzi et al. (2000) 2nd exper. | Yes | 8 | 3.09 | 2.27 (n.s.) | -26.5 | 1.64 | 0.81 (*) | -50.8 |
| (f) Brizzi et al. (2009) | Yes | 8 | 2.26 | 1.62 (x) | -28.2 | 1.01 | 0.91 (n.s.) | -10.0 |
| Wheat growth | | | | | | | | |
| (g) Betti et al. (1997) | Yes | 8 | 1.79 | 1.91 (n.s.) | +7.0 | 0.85 | 0.67 (n.s.) | -21.3 |
| (h) Brizzi et al. (2005) | Yes | 3 | 3.48 | 2.46 (*) | -29.4 | 1.03 | 0.74 (n.s.) | -28.0 |
| Tobacco/TMV | | | | | | | | |
| (i) Betti et al. (2003) | Yes | 3 | 57.52 | 49.83 (**) | -13.4 | 17.06 | 11.69 (n.s.) | -31.5 |

Legend: n = number of experiments; the *p* values are related to comparisons with control; n.s. = not significant; x = $p \leq 0.10$; *$p \leq 0.05$; **$p \leq 0.01$.

Data for this table are taken from an earlier publication [65].

**TABLE 6**
**Effect Size "d" Computed for the Three Plant Models**

| Plant Models | Stress | Effect Size "d" (%) | | |
|---|---|---|---|---|
| | | As 45x vs. C | W 45x vs. C | As 45x vs. W 45x |
| Wheat germination | | | | |
| (a) Betti et al. (1994) | No | 55.56 | — | — |
| (b) Brizzi et al. (2000) 1st exper. | No | 45.11 | 7.52 | 38.46 |
| (c) Brizzi et al. (2000) 2nd exper. | No | 55.06 | 55.06 | 0.00 |
| (d) Brizzi et al. (2000) 1st exper. | Yes | 75.11 | 52.74 | 22.55 |
| (e) Brizzi et al. (2000) 2nd exper. | Yes | 91.43 | — | — |
| (f) Brizzi et al. (2009) | Yes | 48.99 | 32.79 | 17.24 |
| Wheat growth | | | | |
| (g) Betti et al. (1997) | Yes | 38.89 | — | — |
| (h) Brizzi et al. (2005) | Yes | 41.05 | 33.06 | 9.45 |
| Tobacco/TMV | | | | |
| (i) Betti et al. (2003 b) | Yes | 35.01 | 25.67 | 9.21 |

### DISCUSSION AND CONCLUSIONS

The results presented here, together with data collected by other research groups on diseased and healthy plants[48,49], seem to demonstrate that plant model systems can be a useful tool to investigate basic research questions about the effectiveness of homeopathic preparations. Therefore, basic research in homeopathy could move forward by conducting plant studies of high-quality design[66,67] in order to identify specific remedy effects. In fact, botanical bioassays appear suitable for basic experimentation, making it possible to overcome some of the disadvantages of clinical trials, such as placebo effects and ethical difficulties. Moreover, plant-based research relies on a very cheap and almost inexhaustible source of biological material, and it needs a short time for carrying out each experiment and collecting a large sample size, essential for the statistical analysis[68]. In particular, wheat models here presented should be recommended for use in further basic research. It seems worth recalling that, when working in such a particular field of research like homeopathy, it is necessary to apply a thorough statistical analysis, as well as adapting statistical tools to the specific problems that we have to deal with. It would also be extremely desirable to have a repertory of plant diseases, describing the main symptoms, to assist in remedy selection, in addition to a ‘‘*Materia Phytoiatrica*’’ based on provings on healthy plants[48].

As far as our wheat models are concerned, we adopted an isopathic approach because, in previous studies, we observed an "isopathic sensitization", i.e., a considerable increase of homeopathic treatment effects when working with stressed seeds[8]. Referring now to our findings with 45x potency on the *in vitro* germination model, the first general consideration we can make is that the results obtained in our repeated experiments are strongly consistent[7,8,13]. The average number of nongerminated seeds after 4 days of observation is very similar, particularly in the control group, but also satisfactorily so in the As 45x treatment group. This reproducibility seems to suggest a specific effect of As 45x potency and confirms that the *in vitro* wheat germination model may be suitable for further studies on the efficacy of ultramolecular dilutions. As far as W 45x is concerned, the significance of its effect vs. control is less reproducible than As 45x. Although other authors[18,22,23] reported no significant effects of potentized water, the electrochemical behavior of W 45x strongly supports, at least from a physical–chemical point of view[34], the significance indicated by the Poisson test in our experiments. This test fits our wheat germination data very well and allowed us to make some parametric inferences without worrying about population distribution. Moreover, the statistical analysis based on Poisson inference allowed us to demonstrate that the dynamization process is a factor of primary importance for the efficacy of homeopathic treatment. Indeed, dynamization of water itself induces some significant effects when compared with the control.

As far as the wheat growth model is concerned, in the first experiment[15], we observed a curative effect of As 45x on seedling growth. This treatment induced a significant increase in stem length of stressed seedlings compared to the control. A similar effect was confirmed in the second experiment[19], carried out with the same protocol in order to verify whether the same significant results could be obtained working in a different place and with a different experimental team. In the second experiment[19], we were able to apply the Mann-Whitney nonparametric test based on ranks, having almost the same power as the parametric Student's *t* test[15]. We thus repeatedly observed highly significant results, confirming the stimulating effect of homeopathic arsenic. Moreover, the *in vitro* wheat growth model was adopted by other research groups in order to study the reproducibility of the data, a very important aspect in homeopathic research[69]. Actually, the result of a first independent replication trial[18] was a reversal of the original studies[15,19]. As 45x induced statistically significant effects, but inhibited wheat shoot growth instead of enhancing it. In a further reproduction trial[25], the role of three potential confounding factors on the experimental outcome (geographical location of the experiments, influence of the main experimenter, and seed sensitivity to arsenic poisoning) was investigated. The authors did not observe any shoot growth increase after a treatment with As 45x in any of the newly performed experiments; in contrast, the meta-analysis of all 17 experiments performed (including earlier experiments already published) yielded a statistically significant shoot growth decrease and the investigated factors did not seem to be responsible for the effect inversion. Independent reproducibility of basic research in homeopathy remains a difficult issue, and we agree with Binder et al.[18] that the internal and external factors influencing the reaction of the plants to homeopathic potencies still need to be resolved.

As far as tobacco/TMV interaction is concerned, it is a well-known "sensor system" commonly used in basic research in phytopathology[70]. In this model, we used a biotic stress (i.e., TMV infection) instead of a chemical stress (ponderal arsenic in sublethal dose) applied in isopathic wheat models. In this way, we were able to get close to a natural disease system, which could be suitable to represent a general response of living matter to homeopathic treatments. In fact, since the main cell structures and functions are common to the majority of eukaryotes[71,72], plant and eukaryotic microbial bioassays can have potentially relevant implications for therapeutic applications. Since in phytopathology there is no "*Materia Phytoiatrica*" equivalent to the *Materia Medica* for human medicine, there are no standard criteria to guide the selection of the correct remedy and different approaches can be applied[48]. We recall that in our tobacco/TMV model, we applied the *law of similars* to choose the treatment. Our experimental setup, based on the leaf disk as elementary statistical unit, allowed us to collect a large dataset and to average the physiological differences between individual plants. After applying nonparametric tests based on ranks, we observed repeated highly significant results, confirming the curative effect of homeopathic treatments (in particular As 45x), which increased tobacco resistance to TMV.

Furthermore, the specific interest given to statistical methods allowed us to consider variability as one of the targets of homeopathic treatment action[16,65]. We found a regular trend in variability decrease in both its components, "within" and "between" experiments. A similar effect on variability "between" experiments has also been detected in the first reproduction trial of the wheat growth model[18], but not in the second[25]. In any case, we decided to follow this double approach (based on mean effect and variability) for data evaluation when carrying out basic research in complementary medicine, as also done in other research works[22].

Finally, the size effect evaluation seems to confirm the regularity of the efficacy of potentized arsenic and, at a slightly lower level, also of potentized water, pointing out the relevance of the dynamization process for the efficacy of homeopathic treatments[73].

### ACKNOWLEDGMENTS

The authors thank Laboratoires Boiron srl for its collaboration and the grant awarded to one of the authors, Dr. Grazia Trebbi. In particular, the authors wish to thank Dr. Silvia Nencioni and Dr. Luigi Marrari for their advice and cooperation. Additional material support was provided by Weleda Italia srl for statistical analysis, and by Wala Italia srl for medical contribution. The sponsors had no influence whatsoever upon design, conduct, evaluation, and manuscript of this investigation.
